# Performance of self-reported HIV status in determining true HIV status among older adults in rural South Africa: a validation study

**DOI:** 10.7448/IAS.20.1.21691

**Published:** 2017-07-18

**Authors:** Julia K. Rohr, F. Xavier Gómez-Olivé, Molly Rosenberg, Jennifer Manne-Goehler, Pascal Geldsetzer, Ryan G. Wagner, Brian Houle, Joshua A. Salomon, Kathleen Kahn, Stephen Tollman, Lisa Berkman, Till Bärnighausen

**Affiliations:** ^a^ Center for Population and Development Studies, Harvard University, Cambridge, MA, USA; ^b^ MRC/Wits Rural Public Health and Health Transitions Research Unit (Agincourt), School of Public Health, Faculty of Health Sciences, University of the Witwatersrand, Johannesburg, South Africa; ^c^ INDEPTH Network, Accra, Ghana; ^d^ Department of Epidemiology and Biostatistics, Indiana University School of Public Health-Bloomington, Bloomington, IN, USA; ^e^ Department of Global Health and Population, Harvard T.H. Chan School of Public Health, Harvard University, Boston, MA, USA; ^f^ Department of Medicine, Beth Israel Deaconess Medical Center, Harvard Medical School, Boston, MA, USA; ^g^ School of Demography, Australian National University, Canberra, Australia; ^h^ CU Population Center, Institute of Behavioral Science, University of Colorado at Boulder, Boulder, CO, USA; ^i^ Umeå Centre for Global Health Research, Division of Epidemiology and Global Health, Department of Public Health and Clinical Medicine, Umeå University, Umeå, Sweden; ^j^ Department of Social and Behavioral Sciences, Harvard T.H. Chan School of Public Health, Harvard University, Boston, MA, USA; ^k^ Department of Epidemiology, Harvard T.H. Chan School of Public Health, Harvard University, Boston, MA, USA; ^l^ Africa Health Research Institute (AHRI), Mtubatuba, South Africa, KwaZulu-Natal; ^m^ Institute of Public Health, University of Heidelberg, Heidelberg, Germany

**Keywords:** Validation study, South Africa, HIV status, self-report, older adults, public health

## Abstract

**Introduction**: In South Africa, older adults make up a growing proportion of people living with HIV. HIV programmes are likely to reach older South Africans in home-based interventions where testing is not always feasible. We evaluate the accuracy of self-reported HIV status, which may provide useful information for targeting interventions or offer an alternative to biomarker testing.

**Methods**: Data were taken from the Health and Aging in Africa: A Longitudinal Study of an INDEPTH Community in South Africa (HAALSI) baseline survey, which was conducted in rural Mpumalanga province, South Africa. A total of 5059 participants aged ≥40 years were interviewed from 2014 to 2015. Self-reported HIV status and dried bloodspots for HIV biomarker testing were obtained during at-home interviews. We calculated sensitivity, specificity, positive predictive value (PPV) and negative predictive value (NPV) for self-reported status compared to “gold standard” biomarker results. Log-binomial regression explored associations between demographic characteristics, antiretroviral therapy (ART) status and sensitivity of self-report.

**Results**: Most participants (93%) consented to biomarker testing. Of those with biomarker results, 50.9% reported knowing their HIV status and accurately reported it. PPV of self-report was 94.1% (95% confidence interval (CI): 92.0–96.0), NPV was 87.2% (95% CI: 86.2–88.2), sensitivity was 51.2% (95% CI: 48.2–54.3) and specificity was 99.0% (95% CI: 98.7–99.4). Participants on ART were more likely to report their HIV-positive status, and participants reporting false-negatives were more likely to have older HIV tests.

**Conclusions**: The majority of participants were willing to share their HIV status. False-negative reports were largely explained by lack of testing, suggesting HIV stigma is retreating in this setting, and that expansion of HIV testing and retesting is still needed in this population. In HIV interventions where testing is not possible, self-reported status should be considered as a routine first step to establish HIV status.

## Introduction

The HIV epidemic in South Africa has vastly changed over the past decade with the expansion of testing, treatment and prevention programmes, leading to increases in life expectancy, less HIV transmission and improved economic productivity [1–6]. Yet, an estimated 24% of HIV-positive South African adults are undiagnosed [7]. HIV prevention interventions are often de-coupled from testing, but HIV status information is important to effectively target interventions to people who will benefit from them. For example, treatment initiation and adherence support, treatment-as-prevention strategies, medical male circumcision, pre-exposure prophylaxis and HIV counselling programmes all benefit from knowing an individual’s HIV status. In population-wide initiatives, self-reported status may be a useful source of information.

Older adults have been neglected in HIV testing initiatives, which have traditionally focused on adolescents and reproductive-age adults <50 years old [2,3,8–10]. Older adults in sub-Saharan Africa have less knowledge and awareness of HIV and are less likely to be tested [7,11–13]. Over 40% of HIV-positive South Africans aged 60 years and older do not know their status [7], yet older adults make up a substantial and growing proportion of people living with HIV in South Africa [3,14–17]. They are at risk of HIV acquisition and present unique challenges to HIV care, such as more rapid disease progression, slower antiretroviral therapy (ART) response and complications from cardiometabolic disease co-infections and cognitive decline [2,8,9,17–35]. More HIV programmes are needed to target older populations, who are likely to be reached in home-based interventions where biomarker HIV testing is not always feasible. Self-reported HIV status may provide useful information to target interventions or offer an alternative to biomarker testing [36], though few studies have examined its validity in sub-Saharan African populations. In population-based surveys of adults in Kenya (2012 to 2013), Uganda (2011) and Malawi (2010), asking HIV status was feasible though self-report was somewhat low (26–47% of the HIV-positive population) partly due to lack of HIV testing or repeat testing [37,38]. The validity of using self-reported status as a proxy for biomarker-confirmed status among older adults in sub-Saharan Africa has not been examined.

In the ongoing Health and Aging in Africa: A Longitudinal Study of an INDEPTH Community in South Africa (HAALSI) study, we have the unique opportunity to investigate the validity of self-reported HIV status among older adults in rural South Africa using biomarker HIV status as the “gold standard”. We establish for the first time the positive predictive value (PPV) and negative predictive value (NPV), sensitivity and specificity of HIV status self-report. We also assess who is most likely to accurately report their HIV status and what factors influence accurate self-report.

## Methods

### Study population and data collection

We analysed data from the HAALSI cohort baseline survey, conducted from November 2014 through November 2015 to investigate the health and well-being of the older population in South Africa. Participants were sampled from all individuals 40 years and older and permanently living in the Agincourt Health and Socio-Demographic Surveillance System (Agincourt HDSS) site in rural Mpumalanga province [39]. We selected a total of 6281 people, of which 315 were deceased and 76 had moved out of the study site. Of the remaining sample, 86% (5059) participated in the survey, while 7.3% refused, 6.0% were not found and 0.8% were unable to participate. Future survey rounds are planned to study changes in health and well-being of cohort members as they age. Extensive survey and biomarker data were collected during in-person interviews by trained local fieldworkers, who used Computer Assisted Personal Interviews to assess physical and cognitive functioning, cardiometabolic health, economic well-being and HIV. Ethical approval for HAALSI was obtained from the University of the Witwatersrand Human Research Ethics Committee, the Harvard T.H. Chan School of Public Health Office of Human Research Administration and the Mpumalanga Provincial Research and Ethics Committee. All participants provided informed consent prior to being interviewed.

HIV and ART status were obtained through both self-report and biomarker testing at time of the interview. Fieldworkers collected blood via finger prick and prepared dried bloodspots (DBS). HIV screening and confirmatory enzyme-linked immunosorbent assays (ELISA) were done using the Vironostika HIV 1/2 Ag/Ab MicroELISA System (BioMérieux, Marcy-l’Étoile, France) and the Roche Cobas E411 Combi Ag, respectively. DBS were also screened for presence of ART through testing for lamivudine (3TC) and emtricitabine (FTC), which are the standard second drugs in both first- and second-line three-drug regimens in South Africa. Study samples were analysed at the Pharmacokinetic Laboratory at the University of Cape Town. Presence of either drug >0.02 µg/mL was considered positive for ART.

HIV testing and status were queried through the following questions with no skip pattern: (1) “Have you ever been tested for HIV?”, (2) “When was the last time you had an HIV test?”, (3) “Do you know your HIV status?” and (4) “Have you ever tested positive for HIV?”. We evaluated the predictive value of question (4). Covariates were similarly obtained through interview responses and included age, sex, education, employment, marital status and household wealth. A wealth index was created based on household characteristics and ownership of household items, vehicles and livestock [40].

### Analysis

We examined the proportion and characteristics of participants who consented to DBS biomarker testing compared to those who did not and used chi-square (*Χ*^2^) tests. The analysis was limited to participants who consented to biomarker testing and had valid test results. Responses to “Have you ever tested positive for HIV?” were compared to the “gold standard” biomarker test result. We calculated the question’s PPV (proportion reporting testing HIV positive who were truly positive), NPV (proportion reporting not testing HIV positive who were truly negative), sensitivity (proportion who were truly HIV positive who reported testing HIV positive) and specificity (proportion who were truly HIV negative who did not report testing HIV positive) [41,42]. These parameters were also calculated among those who reported ever having an HIV test, among those who report knowing their HIV status, and stratified by age group and sex.

Our secondary analysis examined factors associated with accurate self-report of HIV-positive status. Demographic variables and ART status were considered as predictors of sensitivity of self-report among those who tested HIV positive, using crude and age- and sex-adjusted log-binomial regression models [43].

## Results

**Figure 1.**
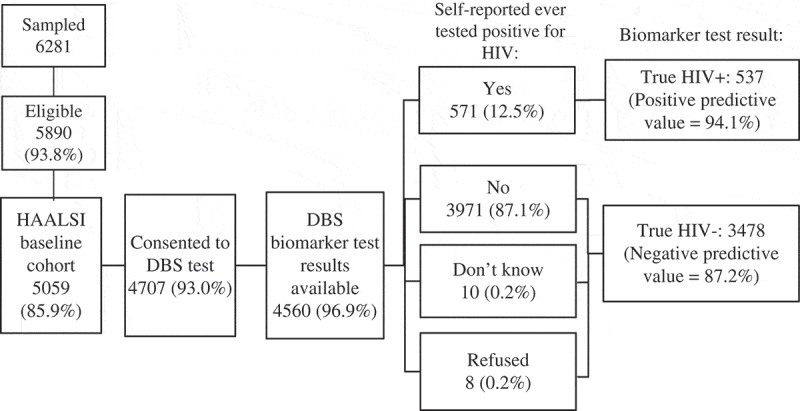
HAALSI baseline survey responses to dried bloodspot (DBS) biomarker testing and HIV self-report.

In the HAALSI baseline survey, 4707 (93%) of the 5095 respondents consented to DBS biomarker testing. Valid results were available for 4560 individuals and were included in the analytic sample (Figure 1). Of those with invalid results, 22 had an indeterminate HIV test result, and the remainder had either poor quality or not enough spots collected. The proportions consenting to biomarker testing were similar among females (93.4%) and males (92.4%) and were over 90% for all ages. Consent was slightly higher among those who reported ever having an HIV test (94.2% vs. 91.1%, *Χ*^2^ *p* < 0.001) and among those who reported being HIV positive (95.0% vs. 92.8%, *Χ*^2^ *p* = 0.03).

Out of the 4560 individuals with biomarker results, 64.7% reported ever testing for HIV and 58.4% reported knowing their status (Table 1). All respondents were asked if they ever tested positive for HIV regardless of their responses about HIV testing, giving an overall self-reported HIV prevalence of 12.5%. Measured biomarker HIV prevalence was 23.0%. Anyone who reported never having tested positive for HIV, refused or “did not know” was considered to be self-reported HIV negative in analysis (Figure 1).

**Table 1.** Study population characteristics

Population	*N* (% of population)	Reported ever had an HIV test (%)	Reported being HIV positive (%)	Biomarker tested HIV positive (%)
Total	4560	64.7	12.5	23.0
Reported ever had an HIV test	2949 (64.7)	100	19.1	28.7
Reported knowing their HIV status	2665 (58.4)	97.9	21.0	30.0
Gender				
Males	2097 (46.0)	63.1	12.6	23.0
Females	2463 (54.0)	66.0	12.5	22.9
Age (years)				
40–49	814 (17.9)	74.5	19.7	38.1
50–59	1262 (27.7)	73.3	18.3	31.1
60–69	1203 (26.4)	64.7	10.8	20.3
70–79	808 (17.7)	54.0	5.6	11.0
80+	473 (10.4)	43.1	1.1	2.8

We found that half of those with biomarker results (50.9%) reported knowing their status and accurately reported it. Overall, PPV of self-report was 94.1% (95% confidence interval (CI): 92.0–96.0), NPV was 87.2% (95% CI: 86.2–88.2), sensitivity was 51.2% (95% CI: 48.2–54.3) and specificity was 99.0% (95% CI: 98.7–99.4). Restricting the analysis to those who reported ever testing for HIV or those who reported knowing their HIV status did not improve the predictive value, although the sensitivity increased to 63.2% and 66.2%, respectively (Table 2).

**Table 2.** HIV self-report performance criteria

Population	**PPV** (95% CI) (%)	**NPV** (95% CI) (%)	**Sensitivity** (95% CI) (%)	**Specificity** (95% CI) (%)
Total	94.1 (92.0–96.0)	87.2 (86.2–88.2)	51.2 (48.2–54.3)	99.0 (98.7–99.4)
Subgroups				
a. Reported ever had an HIV test	94.9 (93.0–96.7)	86.9 (85.6–88.3)	63.2 (59.9–66.4)	98.6 (98.1–99.1)
b. Reported knowing their HIV status	94.6 (92.8–96.4)	87.2 (85.8–88.6)	66.2 (62.9–69.5)	98.4 (97.8–99.0)
c. Gender				
Males	94.3 (91.5–97.1)	87.2 (85.7–88.8)	51.6 (47.1–56.0)	99.1 (98.6–99.5)
Females	93.8 (91.1–96.5)	87.2 (85.7–88.6)	51.0 (46.9–55.1)	99.0 (98.6–99.5)
d. Age (years) 40–49	96.3 (93.3–99.2)	76.2 (72.9–79.4)	49.7 (44.1–55.2)	98.8 (97.9–99.8)
50–59	93.5 (90.3–96.7)	82.9 (80.6–85.2)	55.1 (50.2–60.0)	98.3 (97.4–99.1)
60–69	93.9 (89.7–98.0)	88.6 (86.7–90.5)	50.0 (43.7–56.3)	99.2 (98.6–99.7)
70–79	91.1 (78.8–97.5)	93.7 (92.0–95.4)	46.1 (35.7–56.4)	99.4 (98.6–99.9)
80+	80.0 (28.4–99.5)	98.1 (96.8–99.3)	30.8 (9.1–61.4)	99.8 (98.8–100)

PPV: positive predictive value; NPV: negative predictive value; CI: confidence interval.

Predictive values were equivalent among males and females but varied with age (Table 2). PPVs were lower and NPVs were higher in older age groups, reflecting the lower HIV prevalence. Sensitivity among 40–49-year-old women was slightly higher (53.3%; 95% CI: 45.7–60.8) than among men (45.4%; 95% CI: 37.2–53.6), possibly related to antenatal testing. Yet, the predictive values in the 40–49-year-old age group did not differ substantially by sex (PPV for females was 95.7% (95% CI: 89.5–98.8) and males was 97.0% (95% CI: 89.5–99.6); NPV for females was 77.9% (95% CI: 73.6–82.2) and males was 74.1% (95% CI: 69.1–79.1)).

In the sub-analysis that explored predictors of correct self-report, regression models showed that there was no difference in sensitivity between males and females or any trends by education, employment status or wealth index. Sensitivity was slightly lower among older age groups (Supplementary Table 1). Notably, individuals who screened positive for ART were more likely to report their HIV-positive status. The age- and sex-adjusted prevalence ratio for correct self-report for participants on ART vs. not on ART was 2.66 (95% CI: 2.19–3.23) among individuals reporting having taken an HIV test. The ART screening results also show that out of the 48.8% of the HIV-positive population that does not report their HIV-positive status, 41% are on ART, providing a minimum estimate for the proportion who knowingly misreport their status.

**Figure 2.**
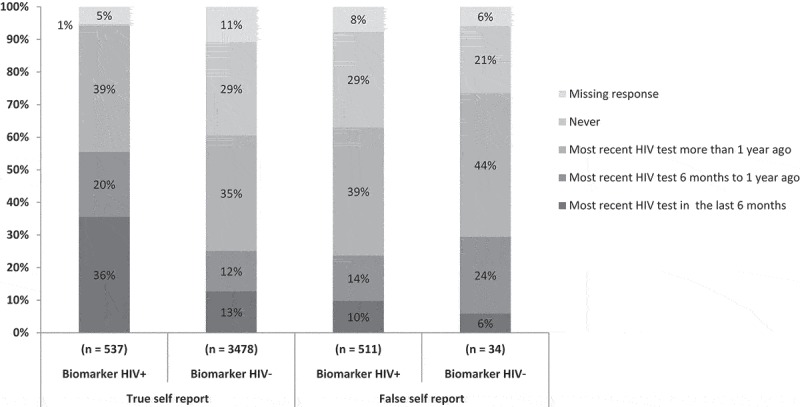
Time since most recent HIV test, stratified by accuracy of self-reported status and biomarker HIV status.

Lastly, we considered time since last HIV test as a possible explanation for why some HIV-positive individuals may incorrectly report being HIV-negative because respondents may have acquired HIV during the time since testing. We found that among all HIV-positive individuals, those who correctly reported being HIV positive had more recent HIV tests (56% reported testing in the past year) than those who incorrectly reported being HIV negative (24% reported testing in the past year) (Figure 2).

## Discussion

In a population-based sample of older adults in rural South Africa, we found that self-reported HIV status was highly predictive of biomarker-confirmed HIV status. This result has important programmatic and policy implications as HIV testing, prevention and care management are increasingly needed and targeted towards older South Africans. Asking HIV status in in-person interviews was also feasible, and only 8 out of 4560 individuals refused to answer the question about HIV status.

The high PPV of self-reported HIV status (94.1%) indicates that individuals reporting being HIV positive are almost certainly HIV positive and should be referred to counselling for preventing HIV transmission and to determining ART status for linkage to care. Individuals reporting being on ART who can demonstrate their ART status may not need further testing. If an individual reports not being on ART, they may be linked to post-test counselling while confirmatory HIV testing is done. HIV care programmes may save resources by using information from self-reported status before running unnecessary tests and may cut down on patient loss to follow-up by not waiting for biomarker testing before referring to care.

The NPV of self-reported HIV status is also high at 87.2%, but there is a >10% chance that an individual who reports being HIV negative is truly positive. Thus, while individuals reporting being HIV negative are likely truly negative and should be referred to counselling for HIV prevention, follow-up HIV testing is still needed for these individuals. HIV prevention counselling can discuss risk of HIV acquisition among older adults and promote the importance of testing or retesting.

Asking additional questions about previous HIV testing did not improve the predictive value of self-report, as both PPV and NPV remained essentially the same when limiting the population to individuals who reported knowing their status or to those who reported ever having been tested. Thus, even if an individual reports being HIV negative and says they know their status or have been tested for HIV previously, follow-up testing is still needed. We saw differences in predictive values by age, largely due to the lower prevalence of HIV among older age groups. Adults ≥80 years old were more likely to correctly report being HIV negative (NPV: 98%) or incorrectly report being HIV positive (PPV: 80%), and confirmation of HIV-positive reports may be more important for these individuals. We found no difference by sex, which was unexpected because in younger age groups men are typically less likely to test for HIV and to link to care than women [10,38,44].

While most HIV-positive individuals can be identified through self-reports, a large proportion of HIV-positive older adults (51%) remain unidentified through self-report, and expanding testing to reach older adults is essential. False-negative reports could be explained by not having tested for HIV, having an outdated HIV-negative test result or knowingly misreporting. A sensitivity of 66% among those who report knowing their status is high, considering the historically high prevalence of HIV-related discrimination in South Africa, and stigma may decrease further as testing becomes more commonplace and ART use expands [45–47]. Being on ART was associated with higher sensitivity of self-reported HIV status, indicating that ART use facilitates disclosure and discussion of positive HIV status..

Our estimate for sensitivity of self-reported HIV status was higher than studies from earlier years that have evaluated self-report among sub-Saharan African adults. The Malawi demographic and health survey from 2010 found 26% sensitivity of self-report for men and 39% for women; the Uganda AIDS Indicator Survey (AIS) from 2011 found 32% sensitivity for men and 44% for women; and the Kenya AIS from 2012 to 2013 found 47% sensitivity overall [37,38]. Though these surveys were conducted among younger adults and in different countries, sensitivity may improve with time and more widespread HIV testing. Testing initiatives, including home-based and repeat HIV testing, have been increasing in recent years in South Africa, leading to improved testing coverage [7,48,49]. Our results indicate that increased testing and retesting are still needed among older South Africans for both males and females to improve sensitivity of self-report and to make progress towards reaching the UNAIDS 90-90-90 targets [50], which are not yet being met in this population based on self-report of testing and awareness of status.

Our estimates for the predictive value of self-report are likely representative of the study population. Even though individuals who were already aware of their HIV status appeared to be more comfortable consenting to biomarker testing, the consent rates were high (>90%) among all segments of the population. Our study was limited by only evaluating the predictive value of the question “Have you ever tested positive for HIV?” whereas a question phrased differently, such as “What is your HIV status?”, could produce more false-positive reports.

Asking self-reported HIV status was feasible in this population, and the majority of participants were willing to disclose their HIV status to local field workers. Our estimates of sensitivity and specificity can assist researchers and policymakers who have self-reported HIV information to estimate HIV prevalence. When reaching older adults in settings where biomarker testing is expensive or impractical, our estimates for predictive values give insight into how to best approach HIV interventions. While testing efforts are still needed, use of self-reported status should be considered as a low-cost first step to effectively target HIV programmes and interventions for older South Africans.

## Abbreviations

3TC, lamivudine; AIS, AIDS Indicator Survey; ART, antiretroviral therapy; CAPI, Computer-Assisted Personal Interview; DBS, dried bloodspots; ELISA, enzyme-linked immunosorbent assay; FTC, emtricitabine; HAALSI, Health and Aging in Africa: A Longitudinal Study of an INDEPTH Community in South Africa; NPV, negative predictive value; PPV, positive predictive value.

## Notes

To access the supplementary material to this article please see Supplementary Files under Article Tools online.
